# CD44, Hyaluronan, the Hematopoietic Stem Cell, and Leukemia-Initiating Cells

**DOI:** 10.3389/fimmu.2015.00235

**Published:** 2015-05-26

**Authors:** Margot Zöller

**Affiliations:** ^1^Department of Tumor Cell Biology, University Hospital of Surgery, Heidelberg, Germany

**Keywords:** CD44, hematopoietic stem cells, leukemia-initiating cells, bone marrow niche, homing, adhesion, dormancy, apoptosis resistance

## Abstract

CD44 is an adhesion molecule that varies in size due to glycosylation and insertion of so-called variant exon products. The CD44 standard isoform (CD44s) is highly expressed in many cells and most abundantly in cells of the hematopoietic system, whereas expression of CD44 variant isoforms (CD44v) is more restricted. CD44s and CD44v are known as stem cell markers, first described for hematopoietic stem cells and later on confirmed for cancer- and leukemia-initiating cells. Importantly, both abundantly expressed CD44s as well as CD44v actively contribute to the maintenance of stem cell features, like generating and embedding in a niche, homing into the niche, maintenance of quiescence, and relative apoptosis resistance. This is surprising, as CD44 is not a master stem cell gene. I here will discuss that the functional contribution of CD44 relies on its particular communication skills with neighboring molecules, adjacent cells and, last not least, the surrounding matrix. In fact, it is the interaction of the hyaluronan receptor CD44 with its prime ligand, which strongly assists stem cells to fulfill their special and demanding tasks. Recent fundamental progress in support of this “old” hypothesis, which may soon pave the way for most promising new therapeutics, is presented for both hematopoietic stem cell and leukemia-initiating cell. The contribution of CD44 to the generation of a stem cell niche, to homing of stem cells in their niche, to stem cell quiescence and apoptosis resistance will be in focus.

## Introduction

CD44, first described as a lymphocyte homing receptor (1), is expressed by a wide range of hematopoietic and non-hematopoietic cells (2). Interest in CD44 increased considerably, when it was noted that the insertion of alternatively spliced exon products in the CD44 standard or CD44 hematopoietic isoform (CD44s) strikingly affects the molecules function, such that expression of CD44 variant isoforms (CD44v) induces a metastatic phenotype in locally growing tumor cells (2, 3). At the time, it was surprising that a leukocyte marker is engaged in solid tumor metastasis formation. As the hematopoietic system is the only organ that components repeatedly shift between sessile and mobile states, we argued that metastasizing tumor cells may transiently take over part of the program of hematopoietic cells and that this programmatic shift, which is independent of oncogene transformation, depends to a considerable degree on CD44 and its activities (4). This hypothesis received strong support by the recovery of cancer- and leukemia-initiating cells (CIC/LIC), which are defined by their capacity to take over part of the program of stem cells (SC). In fact, CD44s/CD44v are CIC/LIC markers (5, 6) and, most importantly, CD44 was the first marker defined as a CIC/LIC *bio*marker. This implies that CD44 is engaged in fulfilling special SC-related tasks in CIC/LIC (7). These particular tasks include, besides growth upon serial transplantation in xenogeneic models, self-renewal and recapitulation of the heterogeneous phenotype of the parental tumor, reflecting the differentiation capacity of CIC/LIC. It also includes, at least for a subset of CIC/LIC, the capacity of SC to transiently shift from a sessile toward a mobile state, which is required for metastasis formation (7–9). Furthermore, like SC, CIC/LIC are highly apoptosis resistant (6, 7) and may profit from the crosstalk with the surrounding (5, 6). Notably, too, CIC/LIC are heterogeneous (10) and genetically instable (11). This is in line with their disputed, not mutually exclusive origin from adult stem cells (ASC), from oncogene-transformed committed progenitors or from cell fusion particularly with macrophages (Mϕ) (12). Despite their heterogeneous origin, CIC/LIC share many features with hematopoietic SC (HSC), like relative quiescence, longevity, drug resistance, and support by the surrounding that for SC is called the niche. Finally, there is strong evidence that CD44/CD44v is engaged in many of the activities, which CIC/LIC share with ASC (13).

CD44 is a quite abundant expressed molecule. Thus, the question arises, what qualifies CD44 for this multitude of very special tasks. This review outlines that two features of CD44 mostly account for the molecule’s contribution to SC maintenance: first and most important, CD44 crosstalks with the surrounding/the niche. Second, CD44 is located in membrane subdomains, which are particularly prone for collecting signal transduction molecules, proteases, and cytoskeletal components, and foster concerted activities. HSC and LIC were chosen as prominent examples. Based on the largely overlapping activities of CD44 in CIC and LIC, some references to CIC are included, as far as deeper insight was gained with the latter.

## CD44 Structure and Ligands

CD44 are glycoproteins encoded by a single gene (14). CD44 molecules vary in size due to *N*- and *O*-glycosylation (15, 16) and insertion of alternatively spliced exon products in the extracellular domains of the molecule (17). The smallest, hematopoietic isoform (CD44s) is present on the membrane of most vertebrate cells (3). CD44 has seven extracellular domains, a transmembrane, and a cytoplasmic domain (18). The latter is encoded by exons 9 or 10 (19). Between domains 5 and 6 up to 10, variant exon products can be inserted by alternative splicing (15).

CD44 is a member of the family of cartilage link proteins (15, 16). The *N*-terminal region forms a globular structure. Conserved cysteins are important for the stability of the extracellular domain, and two cysteins in the flanking region account for correct link domain folding (19). This globular structure contains binding sites (AA 32–132) for collagen, laminin, fibronectin (FN), and cell surface receptors like E-selectin and l-selectin (20–22). Importantly, CD44 also is the major receptor for hyaluronan (HA) (23). HA binds to a basic motif (AA 150–158) within the globular structure, but outside of the link domain (23, 24). Though the HA binding motif is present in all CD44 isoforms, not all CD44^+^ cells bind HA. However, HA-binding can be induced by CD44 cross-linking, which indicates that HA-binding depends on conformational changes or a redistribution of CD44 in the cell membrane (25). CD44 also has two binding sites for other glycosaminoglycans (GAG) (26). The *N*-terminal globular domain is followed by a stretch of 46 AA, which comprises exon products 5–7. This stretch of 46 AA forms a stalk like structure (27). It is heavily glycosylated and contains putative proteolytic cleavage sites (28). Variable exon products are inserted in the stalk like region (29). The transmembrane region supports CD44 oligomerisation and contributes to incorporation in glycolipid-enriched membrane microdomains (GEM) (30). The cytoplasmic tail of CD44 contains binding sites for the cytoskeletal proteins ankyrin and ezrin, radixin, moesin (ERM). Ankyrin mediates contact with spectrin and is involved in HA-dependent adhesion and motility (31). ERM proteins are engaged in regulating migration, cell shape, and protein resorting in the plasma membrane (32). The *N*-terminus of activated ERM proteins binds to a motif between the transmembrane region and the ankyrin binding site, and their C-terminus binds to F-actin. Thereby, ERM proteins link CD44 to the actin cytoskeleton (33). Merlin, an additional ERM family member, which lacks the actin-binding domain, might be involved in stabilizing the junctional-cortical actin interface through its *N*-terminal domain (34). The binding of CD44 to cytoskeletal linker proteins influences signaling pathways downstream of CD44, which expands the range of CD44-mediated functions.

Finally, CD44 O-glycosylation, the transmembrane region, and the cytoplasmic tail affect the membrane subdomain localization. Depending on the activation state, CD44 is recruited into GEM (35), which has great bearing on the interaction of CD44 with extracellular ligands and the association with other transmembrane and cytoplasmic molecules (36, 37). These associations are most crucial for the accessory functions of CD44 in migration and signal transduction. This is a sequel of the inner membrane side organization of GEM, which favors harboring adapter and signal transducing molecules like src family members (38). Some of these cytoplasmic adapter and signaling molecules are constitutively associated with GEM-located CD44 (39). GEM are also prone for internalization (40).

Unlike CD44s, CD44v is only expressed on subpopulations of epithelial and hematopoietic cells, particularly during embryonic development, hematopoietic cell maturation and activation, and in some carcinoma and leukemia with a tendency toward overexpression in CIC/LIC (41, 42). Several of the CD44v exon products can contain specific post-translational modifications. These include a heparan-sulfate site in exon v3, which serves for the binding of heparin-binding proteins like basic fibroblast growth factor (bFGF) (43); CD44v6 contains a binding site for hepatocyte growth factor (HGF), vascular endothelial growth factor (VEGF), and osteopontin (OPN) (44–46). OPN also binds to CD44v10 (47). Via these cytokine/chemokine binding sites, CD44v takes over a central and coordinating role in receptor tyrosine kinase (RTK) activation (48).

Briefly, CD44 is the major HA receptor. It has a multitude of additional ligands and associates with transmembrane and cytoplasmic molecules. This is due to several glycosylation sites, variant exon product sequences, the insertion into GEM, and the cytoplasmic tail structure. Noteworthy, HA binding contributes to the GEM recruitment of CD44. Beyond forcing CD44 associations, this has bearing on CD44 internalization.

## HSC/LIC CD44 and Stem Cell Genes

CD44 is a marker of ASC, including HSC and of a large range of CIC and LIC (7, 49). In addition, there is some evidence for pronounced CD44v expression in SC (50). Thus, the question arose, whether CD44 is engaged in stem cell gene expression and/or whether CD44/CD44v expression is regulated by stem cell genes.

Embryonic SC (ESC) are characterized by expression of a set of master SC transcription factors, Oct4, Sox2, and Nanog (51), as well as distinct chromatin organization and epigenetic signatures, which govern the intrinsic ability to self renew and to differentiate into multiple lineages (52). Polycomb genes, which have a role in transcriptional repression through histone modification, associate with the promoter and regulatory regions of target genes in ESC. Expression of master gene transcription factors and epigenetic regulation are maintained in HSC and LIC (53, 54), which share with ESC Oct4, Nanog, and Myc overexpression (55, 56), and Notch, Wnt, and Hedgehog signaling pathways, important in shaping tissue structure, cell fate, and identity (57). In fact, leukemia recurrence was prevented by deletion of the polycomb gene *Bmi1*, important for HSC self-renewal (58).

As discussed below in concern of HSC quiescence, there are links between CD44, Nanog, and Myc expression, and CD44 is a target of the Wnt and Notch pathways in HSC and LIC (59–61). However, there is no evidence that CD44 plays a central role in regulating master gene transcription factors in HSC/LIC (62–64).

Besides master SC transcription factors, miRNA were recognized as key regulators of self-renewal and SC fate (65). This includes hematopoiesis, HSC being lost upon abrogation of Dicer, which was ascribed to miR-125a (66). Additional miRNA overexpressed in HSC either promote HSC engraftment (miR-125b-5p, miR-126-3p, miR-155) or are disadvantageous (miR-196b, miR-181c, miR-542-5p, let7e) (67). Notably, miRNA profiles differ significantly between HSC and lineage committed progenitors, and miRNA profiles in hematological malignancies differ from those of HSC and progenitor cells. In addition, miRNA profiles are selective for distinct leukemia [review in Ref. (68–72)]. Nonetheless, there are some common trends: miR-15a, miR-29b, miR-34a, miR-151, and miR-204 frequently act as tumor suppressors, and miR-155, miR-96, miR-24, miR-21, miR-32, miR-106-25, and let-7 as oncomir (73). However, the engagement of HA/CD44 on miRNA regulation in HSC and LIC remains to be elaborated in detail. So far, there are only sporadic hints toward a mutual impact.

HA-crosslinked CD44v3 binds Nanog, Oct4, and Sox2, which promotes miR-302 expression (74) a key player in controlling SC self-renewal and pluripotency (75). Also, binding of HER2 to CD44 leads to upregulation of MTA-1 (metastasis-associated-1), which induces silencing of the miR-139 promoter, accompanied by increased CXCR4 expression (76). HA-CD44v6 binding promotes PKCε activation, and this increases Nanog phosphorylation and nuclear translocation, where Nanog associates with Drosha and an RNA helicase p68, which leads to oncogenic miRNA-21 transcription and a reduction in the expression of the tumor suppressor programmed cell death 4 (PCD4) (77). CD44v6-associated overexpression of miR-21 (78) induces pre-B-cell lymphoma (79), and is frequently observed in CML (80). Analyzing the impact of CD44v6 on the miRNA profile in metastasizing CIC (81) revealed CD44v6-dependent downregulation of the tumor suppressors let-7b, let-7d, let-7e, miR-101, and miR-34a. The latter, which suppresses tumor growth by CD44 downregulation (82), is abundantly expressed in CD44v6 knockdown (CD44v6^kd^) cells, which argues for CD44v6 to be engaged in miR-34a silencing. On the other hand, metastasis-promoting miR-494 and miR-21 and apoptosis-regulating miR-24-1 (83–85) are abundant only in CD44v6-competent cells. miRNA transcription and/or posttranscriptional regulation also were affected by CD44v6-associated MET (86), which supports miR-103 transcription (87). MiR-103 expression was only high in CD44v6-competent cells.

We are also far away from a comprehensive view on the regulation of CD44 via miRNA. MiR-199a binding to the CD44 3′-UTR suppresses tumorigenicity, multidrug resistance, and migration (88, 89). The CD44 3′-UTR binds additional miRNA that target extracellular matrix (ECM) mRNA, like miR-328, miR-491, miR-671, and miR-512-3p. In fact, transfection-induced CD44 3′-UTR overexpression is accompanied by collagen I and FN upregulation (90). However, stressing the need for further studies, opposing findings have also been reported, such as downregulation of CD44 by pro-metastatic miR-373/520c (91).

Finally, aberrant and alternative splicing is frequently observed in CIC/LIC, and CD44 ranks first in the affected genes (92). However, no mutations were found in *cis* acting CD44 splice elements (93). Thus, a genetic basis for CD44 alternative splicing in malignancies remains questionable.

Taken together, though links between CD44 and master SC genes, dominating SC signaling pathways, and epigenetic regulation of SC genes were described, HSC do not essentially depend on CD44. This could have been expected, as HSC are not or not seriously affected in panCD44^ko^ (94), CD44v10^ko^ (95), CD44v7^ko^, or CD44v6/v7^ko^ (96–98) mice.

On the other hand, it is already known since 1990 that CD44 is required for the development and maintenance of early hematopoietic progenitors. In long-term bone marrow (BM) cultures, tightly packed clusters of small cells, so called cobble stone areas, develop below a stroma layer. These cobble stones contain cells with the capacity for long-term reconstitution. When cultures contain anti-CD44, HSC clusters do not develop (99). Furthermore, CD44 is a reliable LIC marker in many malignancies (100), and the first LIC biomarker that blockade severely affected LIC maintenance, e.g., anti-CD44 drives LIC into apoptosis (101, 102). Thus, the essential contribution of CD44 relies on the communication of SC/HSC and LIC with the surrounding. In the following sections, those features of HSC are discussed that depend on or are modulated by the surrounding. This includes the requirement for a niche to maintain quiescence and to receive signals that drive out of quiescence toward differentiation. The latter frequently is associated with changes in motility. Finally, HSC are relatively apoptosis resistant. It also will be discussed, where LIC, which resemble HSC in many respects, become less dependent on the surrounding or respond differently due to the oncogenic transformation.

## The Endosteal Niche

The fate of a cell in the developing organism is determined by its position (103, 104). SC reside in specialized locations, the niches, which minutely regulate their activity (105). Niches are composed of epithelial and mesenchymal cells and extracellular substrates. They govern location, adhesiveness, retention, homing, mobilization, quiescence and activation, symmetric and asymmetric division, and differentiation (106). Accordingly, a niche might prevent tumorigenesis, which would argue against CIC/LIC profiting from a niche. However, there is ample evidence that a preformed niche supports CIC/LIC survival and homing (105) and regulates the balance between quiescence and growth (107). Beyond this, a niche can support reprogramming of non-CIC toward CIC by exposing them to an embryonic microenvironment (108). CD44 plays a central role in the crosstalk between SC/malignant SC and the niche, which includes an active contribution of CD44 in niche assembly.

### The composition of HSC and LIC niches

A niche for HCS, where they receive instructions particularly in respect to their lifelong capacity for self-renewal, was first proposed by Schofield in 1978 (109). Only 25 years later, it was uncovered that osteoblasts lining the surface of the bone play a major role (110). Additional cellular components of the endosteal niche are mesenchymal stem cells (MSC), osteoclasts, Mϕ, fibroblasts, and adipocytes (111, 112). Interestingly, MSC, too, are influenced by their surrounding. Thus, it was expected that MSC from different tissue fulfill equivalent biological activities. On the contrary, when implanting MSC from BM, white adipose tissue, umbilical cord or skin, only BM-derived MSC spontaneously formed a BM cavity, which was progressively replaced by hematopoietic tissue and bone and permitted homing and maintenance of long-term murine and human HSC (113). Matrix components of the endosteal niche are HA, FN, laminin, and collagen that are secreted by endosteal niche cells and support HSC adhesion, quiescence, and self-renewal. Prominent cytokines and chemokines secreted by BM stroma cells (BM-Str) and/or captured by the BM stroma are thrombopoetin (TPO), SDF1, OPN, and parathyroid hormone. TPO promotes HSC quiescence (114). SDF1 supports quiescence and affects apoptosis resistance (115). OPN is engaged in lodgment to the endosteum (116), and parathyroid hormone supports trabecular network formation of osteoblasts and HSC expansion (117).

Hematopoietic SC avail on a second niche, the vascular niche, which is located in proximity to endothelial cells (118). Though components and activities of the endosteal and the vascular niche are partly overlapping (119), distinct to the endosteal niche, the vascular niche plays a major role in HSC homing and hematopoietic progenitor egress. The vascular niche also supports hematopoietic progenitor expansion and maturation. In line with these special duties, reticular cells in the vascular niche express IL6, HGF, OPN, and SDF1 at high or higher levels than cells in the osteogenic niche (120).

Thus, possibly distinct to ASC in solid organs, HSC dispose of two niches. The requirement for two niches might be linked to the general feature of cells of the hematopoietic system that are not sessile and circulate through the body to fulfill upon request their tasks *in loco*, and thereafter patrol again through the organism. Noteworthy, HSC/LIC CD44 contributes to the establishment of both BM niches.

### CD44 contributes to the generation of the BM niches

#### The Contribution of CD44 to Matrix Assembly

Stem cells niches, including the osteogenic niche in the BM, are particularly rich in HA (121). HSC synthesize and express HA, and HA expression correlates with HSC adhesion to the endosteal niche (122). Similarly, CD44 contributes to building an HA coat on endothelial cells, which facilitates binding of mobilized HSC to endothelial cells as well as HSC homing (123, 124). Furthermore, perturbation in matrix components alters cell shape and intracellular tension, which results in shifts in signaling events that affect gene expression (125). This could be particularly important in the BM niche, where HA delivery by HSC/LIC can induce expression of HA in niche cells (126). Furthermore, CD44 is involved in matrix assembly (127) such that the HA–CD44 association modifies the matrix to support colonization (128).

**Figure 1:**
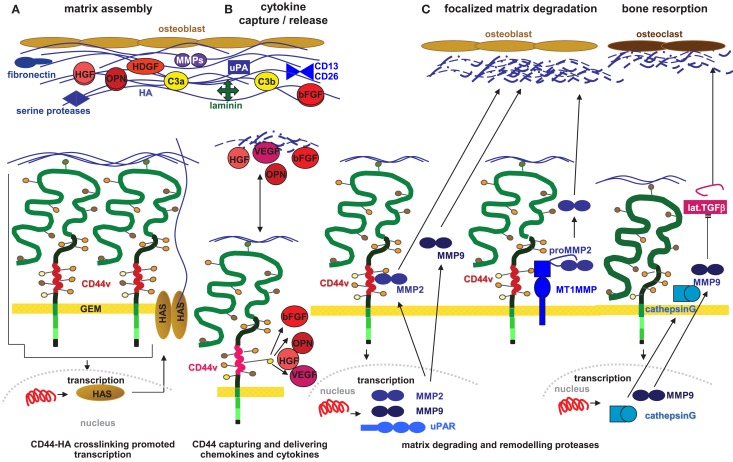
**The contribution of CD44 in HSC/LIC for assembly and modulation of the osteogenic niche**. **(A)** HSC secrete large amounts of HA. Upon HA crosslinking, CD44 becomes activated, which supports transcription of HAS3, which strengthens the process of high MW HA deposition and incorporation of cytokines, chemokines, and proteases in the abundant HA coat. **(B)** HSC quiescence and LIC proliferation are supported by the catcher activity of CD44/CD44v6 and most pronounced CD44v3 associated GAG, which bind a large range of cytokines and chemokines including HGF, bFGF, OPN, and VEGF. **(C)** Particularly, LIC contribute to modulation of the matrix in the osteogenic niche. This is due to CD44-HA initiated transcription of MMP2, MMP9, uPAR, and cathepsinG. MMP2 and MMP9 bind to CD44; proMMP2 is cleaved by CD44v6-associated MT1MMP, which concomitantly allows for focal matrix degradation. CathepsinG and MMP9-activated TGFβ contributes to bone resorption and niche preparation for LIC.

In concern about CD44v, there is evidence for an engagement of CD44v6 in matrix assembly. A CD44v6 knockdown (^kd^) in a highly metastatic tumor line revealed a striking reduction in metastatic capacity, which was, at least, partly due to an altered tumor matrix (81). CD44v6^kd^ cells secrete a matrix not supporting adhesion of CD44v^wt^ or CD44v6^kd^ cells, whereas both cells readily adhere to the CD44v^wt^-matrix. In fact, HA synthase 3 (HAS3) expression is strongly reduced in CD44v6^kd^ cells (86), where high HAS3 expression frequently correlates with aggressiveness of carcinoma and leukemia (129). On the opposite, the CD44v6^kd^ cells abundantly secrete hyaluronidase such that the matrix contains a lower amount of HA and exclusively low molecular weight HA (130), which significantly affects adhesion and the catcher activity of the matrix (Figure 1A).

Briefly, HSC CD44 contributes to the generation of HSC niches mostly via HA provision, where the composition of HA varies depending on the expression of CD44v isoforms. CD44/CD44v strengthens HAS3 expression and, by not yet defined mechanisms, prohibits hyaluronidase activity.

#### CD44 Contributes to the Catcher Activity of the Niche Matrix

CD44 is a transmembrane proteoglycan, which allows for the local concentration of glycosaminoglycan-associating proteins (131). Of special interest for HSC and LIC is the binding of OPN to CD44v3, CD44v6, and CD44v10 (47, 132, 133), where OPN secretion is further stimulated by HA (134). OPN is chemotactic and haptotactic, and as such important for the recruitment of HSC into the niche (135). On the other hand, the OPN–CD44 interaction exerts a feedback on the donor cell, which supports migration. Thus, p53^ko^CD44^ko^ mice have the same rate of primary tumor development as p53^ko^ mice, but tumors do not metastasize (136). Similarly, a blockade of CD44v10 strongly reduced OPN delivery by leukemic cells, which was accompanied by pronounced retention of HSC in the niche (137). CD44v6 also binds VEGF and HGF (44, 45, 138, 139). In the hypoxic environment of the osteogenic niche, HIF1α acts as a regulator to prevent HSC proliferation and exhaustion, where it is supported by VEGF, a target of HIF1α (140). Instead, leukemic cells, which were supported by VEGF-activated endothelial cells in the vascular niche, gain in cytotoxic drug resistance (141). In concern about HGF, it is worthwhile noting that a subpopulation of HSC responds to HGF by migrating toward skeletal muscles (142). CD44v3 binds bFGF that stimulates proliferation of underlying mesenchymal cells in the developing limb and affects BM MSC (143, 144). This might be due to bFGF inducing changes in HAS and hyaluronidase isoform expression (145). A direct contribution of CD44v3-bound bFGF to the activity of bone MSC remains to be explored (Figure 1B).

Finally, CD44v6 can directly contribute to the composition of the niche matrix (130). CD44v6 supports transcription of hepatoma-derived growth factor, which stimulates the growth of fibroblasts, endothelial cells, and vascular smooth muscle cells, and recruits MSC (146). CD44v6 also promotes clusterin secretion that influences chemokine secretion and initiates stromal changes affecting intercellular communications (147). In addition, the complement (C) components 3a and 3b are absent in a CD44v6^kd^ matrix, but are abundantly delivered by CD44v6^wt^ cells (86). These findings are well in line with the innate immune system, particularly C3, cooperating with CD44 in HSC to strengthen the HSC CD44 – niche interaction [review in Ref. (148)] (Figure 1A).

#### CD44 Modulates HSC Niches

CD44 concentrates MMPs at the cell surface, where the production of uPAR, MMP2, and MMP9 is concomitantly stimulated by the interaction between HA and CD44 (86, 149). MMP9 transcription is actively supported by the CD44 intracellular domain (ICD), which binds to a MMP9 promoter response element (150). By a not yet defined mechanism, CD44v6 also is involved in uPAR transcription (86). CD44 aggregation via HA binding facilitates MMP binding (150). Furthermore, proMMP2 becomes activated through CD44v-associated MMP14, which is located in the leading lamella. As cell-bound MMPs are protected from their inhibitors, this allows for focal degradation of the ECM to form space for invading LIC (151). LIC also stimulate osteoclasts to secrete cathepsinG and MMP9 to resorb bone to create a niche. CathepsinG, primarily secreted by osteoclasts (152), is another transcriptional target of HA–CD44 signaling (153). Transforming growth factor β (TGFβ) activation through CD44-associated MMP9 promotes angiogenesis, invasion (154), and enhances osteoclast activity and bone resorption (155) (Figure 1C).

Taken together, HSC require a niche and CD44 contributes to niche assembly. The most prominent CD44 contribution relies on the stimulation of HA provision via pronounced HAS activation. CD44 also supports retaining growth factors and chemokines that are supplied by the different niche elements. This facilitates message delivery from the niche toward the HSC/LIC. There is evidence for a contribution of CD44v particularly in cytokine/chemokine retention. It remains to be explored whether this provides a pronounced profit from the niche for CD44v expressing LIC. CD44 also contributes to modulating the niche by hyaluronidases and proteases that transcription is promoted by CD44 or that become activated via direct or indirect associations with CD44. There is no evidence that LIC contribute to establishing a niche. Rather, LIC are suggested to make use of the HSC niche. It is still disputed whether LIC displace HSC from their niche or actively remodel/destroy the niche (156), such that HSC die by neglect. High hyaluronidase secretion by CD44v6^+^ LIC could favor the latter.

### CD44 supports adhesion, homing, and migration of HSC and LIC

#### CD44, HSC, and LIC Adhesion to the Bone Marrow Stroma

One of the prime functions of the osteogenic niche is the retention of HSC to instruct for longevity and quiescence, which requires firm HSC adhesion. This task is mainly taken over by HA (157). HA binding initiates or, at least, influences most activities of CD44 (158). The importance of CD44 as an adhesion molecule for HSC and LIC has been amply demonstrated (157, 159). The particular engagement of the CD44–HA interaction was confirmed by the finding that HSC adhesion can be blocked by anti-CD44, soluble HA, or hyaluronidase (160).

**Figure 2:**
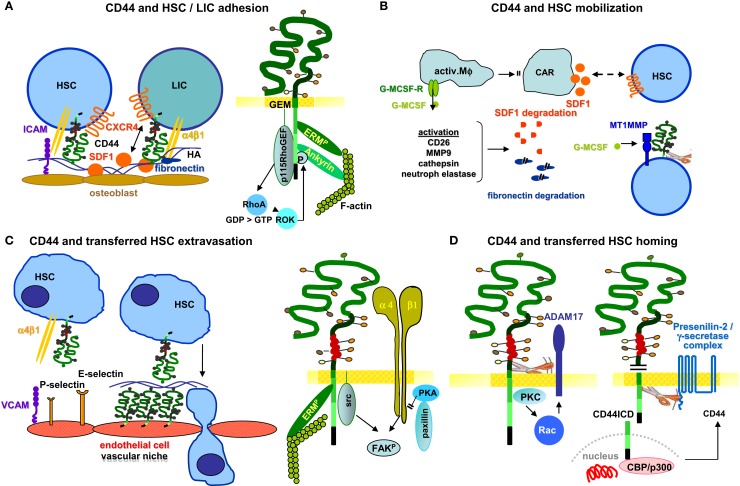
**CD44-mediated adhesion, mobilization, migration, and homing in HSC/LIC**. **(A)** HSC and LIC adhesion to the HA-rich endosteal niche is supported by α4β1, which binds to fibronectin and via ICAM to osteoblasts. CD44-associated CXCR4 binding to stroma-derived SDF1 is of major importance in retaining HSC in the osteogenic niche. One of the adhesion strengthening signaling pathways proceeds via the CD44-crosslinking initiated activation of ERM and ankyrin, which includes activation of a RhoGEF that interacts with RhoA and stimulates ROK. This strengthens ankyrin phosphorylation and the F-actin linkage. **(B)** To interfere with the retention of HSC in the osteogenic niche for PBL-HSC transplantation, adhesion requires to be loosened. This is achieved by blocking CD44 and/or α4β1, and most commonly by G-MCSF that binds to BM-Mϕ, which leads to activation of several proteases that degrade SDF1 and fibronectin. In addition, CAR become attacked and secrete less SDF1. Finally, G-MCSF leads to CD44-associated MT1MMP activation in HSC. By the CD44 cleavage, the adhesion of HSC becomes further reduced. **(C)** Homing of transferred HCS is facilitated by the constitutive expression of P-selectin and l-selectin on BM endothelial cells. CD44 binds to the selectins and α4β1 binds to VCAM-1, which supports loose attachment and rolling. CD44-promoted HA secretion further slows down HSC rolling and allows for extravasation. HA binding initiates activation of CD44, such that it binds to the cytoskeletal linker proteins ankyrin and/or ERM, which link CD44 to F-actin. CD44 moves from the trailing edge to the leading lamella such that the process of extravasation is initiated, where the activation-induced association with integrins plays an essential role. **(D)** It is discussed that the transient cleavage of CD44 by ADAM17 and liberation of the cytoplasmic tail (CD44ICD) by presenilin2/γ-secretase facilitates departure from the endothelium of the BM vasculature. CD44ICD acts together with CBP/p300 as a transcription factor, besides others for CD44. Via this feedback, regain of CD44 allows for HSC adhesion in the osteogenic niche after arrival.

CD44 adhesion to its ligand(s) induces up-regulation of additional adhesion molecules, mostly integrins, which strengthen HSC adhesion (161). This was intensely explored for the association of CD44 with α4β1 (162, 163). Upon activation by HA adhesion, the two molecules directly associate (164), which is accompanied by α4β1-promoted stronger adhesion to FN and laminin (165). HSC α4β1 additionally supports the direct contact with stroma cells via ICAM1 binding (166). In line with the contribution of CD44 and α4β1 to the adhesion of HSC to the osteogenic niche, anti-CD44 and anti-α4β1 dislodge HSC from the BM niche (167, 168) (Figure 2A).

CD44 also participates in LIC embedding into the endosteal niche, such that targeting CD44 is considered a new strategy to eliminate persistent and drug-resistant LIC. In AML, CD44 is required for the transport of LIC to the HSC niche, and anti-CD44 antibodies alter the fate of the LIC by inducing differentiation (101). In a mouse model of CML, BCR-ABL1-transduced progenitors from CD44-mutant donors were defective in BM homing, which resulted in decreased engraftment and impaired CML-like disease induction (100). These studies provided additional evidence that LIC may be more dependent on CD44 for settlement in the osteogenic niche than HSC (101). Studies in a murine model confirmed leukemia cell homing and growth retardation by a CD44-specific antibody (169). However, during reconstitution, a panCD44-specific antibody more efficiently interfered with HSC than leukemia cell settlement in the BM niche (82).

The latter topic, how to avoid replacement of HSC when attacking LIC, was recently approached in an elegant study aiming to find selective ligands for LIC in CML and AML. Alteration of the niche by osteoblastic cell-specific activation of the parathyroid hormone receptor attenuates BCR-ABL1 oncogene-induced CML-like myeloproliferative neoplasia, but enhances MLL-AF9 oncogene-induced AML, possibly through opposing effects of increased TGF-β1 on the respective LIC. These results, though providing a first and very important hint toward sparing niche embedded HSC, also demonstrate that niches differ for distinct LIC (170). The use of CD44v specific antibodies could be an alternative for blocking LIC, but sparing HSC embedding in the niche. Unfortunately, our data so far point toward a dominating role of CD44s in niche embedding. Thus, adhesion of CD44v6/v7^ko^ and of CD44v7^ko^ HSC to BM-Str is unimpaired (97, 171).

Facing competition for the niche, it should be remembered that LIC might remodel the niche such that it no longer serves the requirements of HSC (172). This possibility did not yet receive appropriate attention. Nonetheless, growing awareness and elaboration of the differentiation potential of distinct SC populations, particularly of BM derived MSC, might finally allow reconstituting/replacing a niche, which was distorted by LIC.

Distinct to the contribution of HSC/LIC CD44 on adhesion, little is known on the engagement of stroma cell provided CD44. Anti-CD44v6 strikingly hampered stroma formation in rat long-term BM cultures, but had no impact on HSC embedding in a preformed stroma (173). In addition, recovery of HSC from wt mice in the BM of CD44v7^ko^ mice is severely impaired (97). *In vitro* studies confirmed that HSC poorly adhere to long term CD44v6/v7^ko^ and CD44v7^ko^ BM-Str (171, 174). Thus, BM-Str CD44 should not be neglected as it contributes to matrix assembly. A comprehensive evaluation is missing. However, the availability of conditional ko mice will facilitate answering the question.

There is overwhelming evidence confirming the important finding of Miyake that HSC require CD44 for embedding in the BM niche, and that dislodgement by anti-CD44 severely affects hematopoiesis. In view of the essential role of CD44 to anchor HSC in the osteogenic niche, care should be taken on the therapeutic use of anti-CD44 to drive LIC out of the niche. Despite promising results, further refinement is required to guarantee unimpaired hematopoiesis. First trials to replace anti-CD44 have been successful, but point toward no single strategy being effective in distinct LIC. Additional studies are also needed to elaborate options for correcting niches, which were distorted by LIC.

#### CD44 and HSC Mobilization

Transplantation of hematopoietic progenitor cells provides in many instances an ultimate chance of curative leukemia therapy. It has become obvious that the transfer of peripheral blood HSC appears advantageous, yet it requires HSC mobilization. In 1976, Richman et al. described an increase of HSC in the blood of patients, who had undergone chemotherapy (175). Later, a similar increase was observed after the administration of recombinant growth factors (176). In fact, both G-CSF and chemotherapy mobilize HSC through the same mechanisms, with chemotherapy increasing the level of endogenous G-CSF (177). Though the precise mechanism remains to be elucidated, HSC mobilization obviously does not proceed directly, as HSC do not express the G-CSF receptor, which is expressed by BM macrophages (Mϕ) (178). Activation of Mϕ could result in a reduction of Nes^+^ MSC and SDF1-abundant reticular cells (CAR), and their provision of SDF1 such that the SDF1-CXCR4 bond becomes loosened (179, 180). SDF1 is secreted by several BM-Str and its interaction with CXCR4 on HSC plays a key role in retention and trafficking. CXCR4 expression is enhanced through signaling cascades involving cAMP, PI3K, several GTPases, and PKCζ (181). PKCζ induces motility, adhesion and survival, and MMP2 and MMP9 secretion (182). Disruption of the SDF1-CXCR4 axis is the major mechanism leading to HSC release from their niche (182, 183). Alternatively, not mutually exclusive, upregulated expression of proteases may be involved, which can affect SDF1 (184) via MMP9 (185) or CD26 (186), cathepsin G and K, and neutrophil elastase (183). The same proteases also may account for VCAM1, FN, and OPN degradation (187). In addition, CD44 cleavage via MMP14 can contribute to HSC mobilization, where G-CSF leads to increased MMP14 expression in HSC (188, 189). Activation of the C cascade and plasminogen also contributes to HSC mobilization (190, 191) (Figure 2B).

Although G-CSF efficiently mobilizes HSC in most instances, some patients do not or insufficiently respond. In addition, G-CSF treatment may by accompanied by maturation of the most primitive progenitors, and this impairs HSC homing and recovery of hematopoiesis. Therefore, additional approaches for HSC mobilization have been searched for, in particular, mobilization via a blockade of adhesion molecules expressed by CD34^+^ cells. As described above (167, 168), concomitant application of anti-CD44 and anti-α4β1 most efficiently mobilizes HSC. Notably, most of the mechanisms suggested accounting for G-CSF-induced HSC mobilization might become initiated directly via the CD44 blockade. First, as CD44 is associated with CXCR4, SDF1-CXCR4 binding becomes loosened by a CD44 blockade (192). Second, antibody crosslinking of CD44 contributes to the activation of MMP9 and MT1MMP, which both are associated with CD44 (193).

In brief, mobilization, though mostly approached via G-CSF, can be achieved by directly loosening adhesion of HSC to the niche via anti-CD44 and/or anti-α4β1.

#### CD44 and HSC Homing

With the therapeutic transfer of mobilized HSC, the question arose on their homing. Transferred HSC preferentially home into the BM, where they search for the osteogenic niche (194). Homing is facilitated by the unique feature of BM endothelium that constitutively expresses the endothelial P- and E-selectins and VCAM1 (195). Proinflammatory cytokines stimulate CD44 expression in endothelial cells and strengthen their binding to HA. This promotes the arrest of HSC, which bind via CD44 to HA captured by endothelial cells (196). HSC express P-selectin ligands and CD44 as well as VCAM1 receptors such as α4β1 (197–199). Function blocking antibodies and targeted deletions confirmed the contribution of these adhesion molecules to slow-down HSC, which allows for firm adhesion and extravasation (200, 201) (Figure 2C).

CD44 also is involved in the extravasation of the endothelial cell-attached HSC (202). CD44–HA binding initiates the interaction of the CD44 cytoplasmic tail with the actin cytoskeleton through ankyrin and ERM proteins (196, 203), guiding CD44 to the leading edge of migrating cells (204). Thus, cells expressing CD44 with a truncated cytoplasmic tail retain HA-binding capacity, but loose the capacity to migrate on HA (204). One of the central events in CD44-mediated cytoskeletal reorganization appears to be Rac1 activation. Lamellipodia formation on HA-coated plates can be inhibited by a CD44 blocking antibody, but also by transfection of a dominant-negative mutant form of Rac1 (205). Upstream regulators of Rac1 are Vav1 and Vav2, phosphotyrosine-dependent guanine exchange factors of Rho GTPases. Vav phosphorylation is mediated by src kinases (206). As GEM-located CD44 associates with src (207), cytoskeleton reorganization most likely is initiated via src activation. Another mediator of CD44 signaling is RhoA. The RhoA-specific p115RhoGEF interacts with CD44 and regulates HA-mediated CD44 signaling via the serine-threonine Rho-Kinase (ROK), a downstream target of RhoA. ROK phosphorylates CD44, which promotes enhanced ankyrin binding (208). Another pathway of CD44 promoted motility proceeds via the association with α4β1. By associating with α4β1, CD44 gains access to FAK (focal adhesion kinase) and α4β1 gains access to src kinases and ERM proteins, such that the integrin–paxillin association becomes weakened and the GEM-integrated CD44-ezrin-integrin-FAK complex moves toward the leading edge (164, 209).

Cell motility is additionally supported by CD44 cleavage via a disintegrin and metalloproteinase domain (ADAM) protein and MMP-14 (210). CD44 cleavage is stimulated by Ca^++^ influx, which triggers ADAM10 activation after proADAM10 dissociation from calmodulin. ADAM17, which colocalizes with CD44 at Rac-regulated membrane ruffling areas, becomes activated by PKC and Rac and contributes to CD44v cleavage (211, 212). Thus, the rapid activation of membrane-integrated proteases by CD44–HA binding contributes to a shift toward motility by CD44 cleavage. CD44 cleavage is tightly regulated, in part, by the missing activation of CD44-associated proteases after CD44 cleavage, and in part by cleavage-promoted CD44 transcription. After ectodomain cleavage, CD44 becomes accessible to the presenilin/γ-secretase complex, which triggers intramembrane CD44 cleavage, setting free the CD44 ICD (CD44-ICD). CD44-ICD acts as a cotranscription factor that potentiates CD44 (28), MMP9, and MMP3 transcription (150)(Figure 2D).

As leukemia therapy frequently relies on autologous HSC transplantation, it became important to know whether LIC compete with HSC not only for the niche but also for homing. The described signaling pathways promoting HSC extravasation do not fundamentally differ for LIC. However, there are subtle differences. This was explored in an elegant study for BCR-ABL1-induced CML-like myeloproliferative neoplasia. Expression of α4β1, α5β1, LFA1, and CXCR4 did not differ between BCR-ABL1(+) progenitors and HSC, but expression of P-selectin glycoprotein ligand-1 and of l-selectin was lower than in HSC. Deficiency of E-selectin in the recipient BM endothelium significantly reduced engraftment by BCR-ABL1-expressing SC. Destruction of selectin ligands on leukemic progenitors by neuraminidase reduced engraftment. BCR-ABL1-expressing l-selectin-deficient progenitors were also defective in homing and engraftment, and an l-selectin-specific antibody decreased the engraftment of BCR-ABL1-transduced SC. These results establish that BCR-ABL1(+) LIC rely to a greater extent on selectins and their ligands for homing and engraftment than HSC. Thus, a selectin blockade may be beneficial in autologous HSC transplantation for CML and perhaps other leukemia (213).

After extravasation, transplanted HSC should reach the osteogenic niche. As mentioned, HSC synthesize HA (214), and HA expression supports HSC migration toward the endosteal niche (189). In the endosteal niche, SDF1 promotes adhesion through CD44-associated CXCR4 accompanied by rac1 and cdc42 activation (182, 192, 215). These findings confirm the key role of HA and CD44 in SDF1-dependent HSC anchorage within specific niches (189). Finally, space is created by activated ROK, which phosphorylates the Na-H-exchanger1. Hyaluronidase-2 and cathepsinB become activated in the acidic milieu and support ECM degradation (206, 207).

Unfortunately, AML share the homing mechanisms with HSC (216, 217). However, the problem can possibly be circumvented in leukemia highly expressing CD44v6, as CD44v6 expression is low in HSC and HSC homing is dominated by CD44s. Analyzing migration of HSC from CD44v6/v7^ko^ and CD44v7^ko^ mice toward HA and BM-Str revealed impaired migration toward FN, possibly due to CD44v6 directly binding to FN (218) or being promoted by the CD44v6-α4β1 association (174). Furthermore, HSC migration toward IL6 is strikingly impaired by anti-CD44v6 (219). Migration of CD44v7^ko^ and CD44v6/v7^ko^ HSC toward SDF1 was reduced to background levels, which indicates major importance of these two splice variants in migration along a SDF1 gradient (220). Binding and migration toward OPN is also impaired in CD44v6/v7^ko^ HSC. The finding fits the selective CD44v6 binding of OPN, which triggers migration and invasion (221, 222). Finally, BM-Str CD44v7 supports HSC migration (97). Thus, CD44v6/v7 are engaged in HSC migration toward chemokines/cytokines and BM-Str. Another protein selectively trapped by CD44v6 is C3 (81, 130). As elegantly elaborated by the group of Ratajczak [review in Ref. (223)], C3 can drive CXCR4 into lipid rafts, where it associates with CD44v6. Thereby, the CXCR4–SDF1 axis becomes strengthened, which helps retaining HSC in the niche. In a similar attempt elaborating homing of multiple myeloma to the BM, the authors explored differences in a stroma-dependent and a stroma-independent line. Only the stroma-dependent line expressed IGF-1R and CD44v6, where IGF-1 promoted chemotaxis toward BM-Str and CD44v6 supported adhesion. By modulating the culture conditions, the authors demonstrated that BM-Str promotes up-regulation of IGF-1R and CD44v6 in multiple myeloma, which facilitate homing and support adhesion to BM-Str (224).

Thus, the particular BM endothelium supports the egress of transplanted HSC into the BM. The engagement of CD44 relies on the provision of HA and the binding of CD44 to l-selectin, binding being supported by the association with integrins. Once attached to the endothelium, CD44 promotes activation of rac and rho, which initiate the shift toward a migratory phenotype. Migration is strengthened by activation of CD44-associated proteases. The proteases create space and cleave adhesion molecules including CD44, which fosters migration toward the endosteal niche. Settlement in the niche follows the path that underlies the preferential retention of HSC in the osteogenic niche. In most instances, LIC and HSC use the same adhesion molecules and signaling pathways for migration. Nevertheless and notably, there are some discrete differences between HSC and LIC, which may help elaborating protocols for preferential homing of transplanted HSC.

## CD44 and the Crosstalk between HSC and the Niche

CD44 does not only contribute to niche assembly but, importantly, there is a feedback from the niche toward HSC and LIC, which also involves CD44. Two aspects of this crosstalk will be in focus, the engagement of CD44 in (i) HSC quiescence and (ii) stress resistance, which both are linked to the osteogenic niche. Different to the engagement of CD44 in HSC and LIC homing and migration, mostly HSC rely on CD44-promoted quiescence. Instead, both HSC and LIC profit from CD44 in apoptosis resistance. Though the CD44-mediated crosstalk with the niche and the GEM location of CD44 are important for HSC and LIC apoptosis resistance (225, 226), the dominating mechanisms differ.

In advance of discussing the impact of the niche on CD44-promoted quiescence and apoptosis resistance, exosomes need to be mentioned. Unfortunately, their impact on HSC and LIC has not yet been explored in detail. Many cells including HSC and LIC secrete small vesicles, called exosomes, which are supposed to be most efficient intercellular communicators (227–229), where miRNA transfer via exosomes can lead to target cell reprogramming (230). This was demonstrated for ESC exosomes reprogramming hematopoietic progenitors through miRNA delivery (231), and for the transfer of miRNA between different cells of the hematopoietic system as well as from CIC into BM-Str (232). Although the impact of CD44 on the exchange of exosomal miRNA between HSC/LIC and niche cells has not been elaborated, the impact of exosomal CD44v6 on miRNA transfer points toward the engagement of CD44 (78) and unquestionable demonstrated the strong impact of exosomal miRNA. Thus, a more comprehensive knowledge on the transfer of HCS exosomal miRNA should be approached, and can be expected to open new therapeutic options.

### CD44 and HSC quiescence

The quiescent state is critical for preserving self-renewal capacity and stress resistance of HSC. Besides intrinsic regulatory mechanisms, where p53 plays a dominant role, there are extrinsic microenvironmental regulatory mechanisms, which include angiopoetin-1, TGFβ, bone morphogenetic proteins (BMP), TPO, *N*-cadherin, integrins, Wnt/β-catenin, and OPN (233).

Angiopoietin is secreted by osteoblasts and binds to Tie2 on HSC, which supports maintenance of quiescence and prevents cell division. Furthermore, the Tie2-angiopoietin interaction promotes cobblestone formation in long-term BM cultures (234). Besides strengthening adhesion of HSC to BM-Str (234), possibly via CD44, I am not aware of a particular linkage between the angiopoietin-Tie2 axis and CD44 signaling.

TGFβ are potent inhibitors of HSC proliferation. TGFβ disruption increases circulating progenitor cells, and a bolus injection of TFGβ1 inhibited early progenitor proliferation. TGFβ-mediated quiescence of HSC may be due to alteration in cytokine receptor expression and upregulation of cyclin-dependent kinase inhibitors (235). The engagement of CD44 relies on its interaction with the TGFβR1 (236). TGFβ cooperates with HA-activated CD44 to induce expression of the NADPH oxidase (237), which could help regulating redox signals in HSC.

Bone morphogenetic proteins, secreted by osteoblasts (238), potently inhibit HSC proliferation. BMP bind to their serine threonine kinase receptors on HSC, which leads to transphosphorylation and kinase domain activation, initiating phosphorylation of Smad 1, 5, and 8 that concomitantly associate with Smad4 and translocate to the nucleus. In the nucleus, they act as cotranscription factor regulating expression of target genes such as Runx1 and GATA2, which operate during specification of hematopoiesis (239) and regulate HSC quiescence (110, 240, 241). The linkage to CD44 is based on the association of CD44 with Smad1 (242). Alternatively, and BMP-independent, Smad1 can become phosphorylated via galectin-9, where galectin-9 binding to CD44 promotes formation of a CD44/BMP receptor complex with concomitant BMP receptor activation (243).

Binding of TPO to its ligand (MPL) is critically involved in HSC steady-state maintenance with an over 150-fold reduction of HSC in TPO^ko^ mice. Posttransplantation HSC expansion was highly MPL-and TPO-dependent. Accelerated HSC cell-cycle kinetic in TPO^ko^ mice is accompanied by reduced cyclin-dependent kinase inhibitor *p57^Kip2^* and *p19^INK4D^* expression (244). The activity of TPO becomes strengthened by glucosaminoglycans in the matrix. Though this was demonstrated for megakaryocytopoiesis (245), it may have bearing on TPO affecting HSC embedded in the osteogenic niche.

Wnt signaling has emerged as an important factor in HSC quiescence, self-renewal, and differentiation (246). Wnt, secreted glycoproteins, binds to their sevenpass transmembrane receptors (Frizzled) (247) and low-density lipoprotein receptors LRP5 and LPR6 (248), which become phosphorylated and form an activated Frizzled/LPR receptor complex. The Frizzled/LPR receptor complex promotes dephosphorylation of β-catenin, which in the absence of Wnt signaling is phosphorylated and associated with a so-called destruction complex (249). Dephosphorylated β-catenin translocates to the nucleus, and together with LEF/TCF initiates transcription of Wnt target genes. In the non-canonical pathway, mostly Wnt5a signals via Frizzled using as coreceptor ROR, which leads to RhoA/Rac and JNK activation. In the Wnt–Ca^++^ pathway, G-protein signaling is activated with upregulation of IP3-mediated release of intracellular Ca^++^ and activation of PKC, which triggers nuclear translocation of NFAT and NFκB (246). Though Wnt is a potent morphogen (250), Wnt effects are highly context and dose-dependent (251), which makes it difficult to define precisely its role in HSC maintenance. To circumvent these difficulties, the group of Scadden used an osteoblast-specific promoter for expression of the Wnt paninhibitor Dickkopf1 (Dkk1). Binding to the coreceptor LRP5/6 leads to internalization of the complex (252). Inhibition of Wnt signaling in HSC resulted in reduced p21Cip1 expression, increased cell cycling, and a continuing decline in the reconstitution capacity of HCS. Notably, though the effect on HSC was microenvironment-dependent, HSC did not recover, when transferred in a normal host (253). Furthermore, Wnt-inhibition affected activation of the Notch target, Hes-1. This finding suggests that Notch and Wnt coordinately regulate HSC quiescence. Indeed, elevated Hes-1 and p21 expression correlate with the maintenance of HSC quiescence (254). The importance of Notch signaling was confirmed by inhibition of Notch signaling diminishing the capacity of HSC to maintain an undifferentiated state. As proliferation and survival were not affected, the authors suggest that Notch “may act as a “gatekeeper” between self-renewal and commitment” (255).

Wnt signaling, in fact, is not only important for HSC [review in Ref. (62)], but the association of CD44 with Wnt signaling is also amply demonstrated for LIC [review in Ref. (61)]. However, as mentioned, Wnt effects are context dependent. Highlighting the differences to HSC, one example will be given. The cytoplasmic domain of GEM-located CD44 associates with the Wnt receptor LRP6, whereby LRP6 becomes recruited into the plasma membrane, which strengthens Wnt signaling and the accumulation of β-catenin in the nucleus, where down- and upregulation of CD44 directly affected Wnt signaling. Importantly, this activity of CD44 does not require CD44-HA crosslinking (256).

Osteopontin also negatively regulates the number of HSC in the BM niche. OPN^ko^ mice display a significantly increased number of HSC, but not of committed progenitors (257). OPN also can modify primitive hematopoietic cell number and function in a stem cell–non-autonomous manner. This conclusion derived from the observation that the BM microenvironment of OPN^ko^ mice was sufficient to increase the number of HSC, which was accompanied by an increase in stromal Jagged1 and angiopoietin-1 expression and a reduction of primitive hematopoietic cell apoptosis (116). The authors discuss that the ECM plays a dynamic role in governing HSC responsiveness to expansion signals. Whether the signals are transferred via CD44-associated integrins (258) or directly via OPN binding to CD44 (133) remain to be explored. Instead, OPN binding to CD44v6 in CIC promotes activation of the PI3K/Akt pathway and promotes tumor growth (259). This is mentioned to remember that in concern of signal transduction, the peculiarities of HSC frequently do not allow a direct comparison to oncogene transformed CIC or LIC.

Finally, the interaction of SDF1, expressed by developing stroma in fetal bones, with HSC CXCR4 is critically for retaining HSC in the quiescence-protecting niche (260). Originally, it was noted that SDF1^ko^ and CXCR4^ko^ embryos have greater impairment of myelopoiesis in the BM than in the fetal liver, which suggested that SDF1 and CXCR4 are primarily involved in colonization of the BM by HSC during embryogenesis (261). Later on, elegant work with conditional CXCR4^ko^ mice implicated stromal SDF1 and its receptor in maintaining the pool of quiescent HSC. Conditional CXCR4^ko^ mice have a significantly increased pool of HSC in G1 compared to wt mice. This may be due to an altered environment with upregulation of cytokines, which promote HSC cycling and differentiation (115). Interestingly, actively signaling CXCR4 is associated with GEM localization (262). This implies that CXCR4 signaling sensitivity can be modulated by colocalization with other signaling molecules, including Rac1 (263). Notably, HA-crosslinked, GEM-located CD44 directly interacts with CXCR4, such that SDF1–CXCR4 signaling is abrogated in CD44^kd^ cells (264). Less is known about the impact of the niche on the resting versus cycling state of leukemia. However, it can be expected that due to oncogene transformation LIC are less susceptible to quiescence promoting signals from the niche or may even distinctly respond.

**Figure 3:**
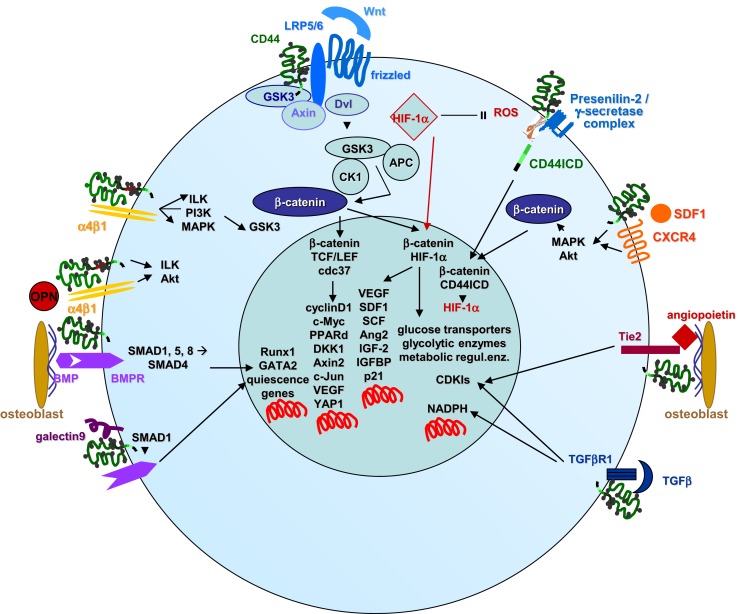
**CD44 imparts quiescence and oxidative stress protection in HSC**. HSC quiescence and oxidative stress resistance are partly cell-autonomous processes, but are supported by a variety of interactions with BM-Str derived factors, where CD44 comes into play by its linkage and activation via HA binding as well as by its association with a variety of growth factor and chemokine receptors such as LRP5/6, CXCR4, Tie2, the TGFβR1, BMPRs, and α4β1. Only those signal transduction pathways and transcription factor activation/nuclear localization are shown, where HSC CD44 plays a major role. Notably, CD44ICD is suggested to be an important cotranscription factor for HIF-1a, the major regulator to cope with the low oxygen in the osteogenic niche.

Figure 3 summarizes those signaling pathways in HSC, where CD44 is actively involved in maintaining HSC quiescence and HSC stress protection. Thus, CD44 becomes stimulated by several key molecules engaged in HSC quiescence. Alternatively, CD44, particularly when crosslinked by HA, associates with quiescence regulating molecules.

Last, not least, the importance of the CD44–HA interaction was also demonstrated with HAS3^ko^ mice. HSC homing into the osteogenic niche depends on the HA coat of endothelial cells, and is significantly retarded in HAS3^ko^ mice (265). Furthermore, HA is required for the generation of HSC during differentiation of ESC (266). Finally, HSC seeded on HA rarely proliferate and retain multipotency (267, 268). All these findings strengthen the upmost importance of the CD44–HA crosstalk in HSC maintenance.

### CD44 and HSC stress resistance

The distinction between HSC and LIC also accounts for a second phenomenon, the response to low oxygen pressure according to the location of HSC in niches (110), characterized by low oxygen concentration (269). Though LIC compete with HSC for the niche, they are not dependent on low oxygen and instead remodel the niche toward accumulation of inflammatory myelofibrotic cells, which drive LIC expansion, but compromise HSC maintenance (270). Notably, too, the metabolic status of HSC residing in a hypoxic BM environment also differs from that of their differentiated progeny (271).

Hematopoietic SC maintains redox homeostasis by low oxygen production due to the minimal metabolic rate (271, 272). Low metabolic rate maintenance further relies on asymmetric cell division, where the daughter cell, which remains in the SC state, inherits a very low level of energized mitochondria (273, 274). Furthermore, HSC generate energy mainly via anaerobic metabolism maintaining a high rate of glycolysis, which limits the production of reactive oxygen species. The hypoxia responsive regulatory pathways in HSC resembles that in other cells, HIF1α being the master regulator driving the metabolic machinery toward anaerobic glycolysis (275). HIF1α, stabilized under hypoxic conditions (276), reprograms glucose metabolism via transcriptional activation of genes encoding glucose transporters, glycolytic enzymes, and metabolic regulatory enzymes, thereby switching from oxidative to glycolytic metabolism (277). One of the mechanisms proceeds via the HSP GRP78 and its ligand Cripto, HIF1α binding to the Cripto promoter (278). The importance of HIF1α orchestrating molecular responses, which maintain redox homeostasis in the face of changing O_2_ levels, was demonstrated by the loss of reconstitution capacity of HSC in HIF1α^ko^ mice (279). HSC dispose on additional regulatory molecules, including polycomb, DNA damage-related, and anti-oxidant proteins that participate in ROS regulation (280). Notably, maintenance of the hypoxic state of HSC is not restricted to the location in a poorly vascularized niche, but is dictated by cell-specific mechanisms derived from their glycolytic metabolic profile (281). Finally, the cotranscription factor CD44–ICD promotes expression of HIF2α (282), as well as of additional hypoxia-related genes, like aldolase c, 6-phosphofructose-2-kinase, pyruvate dehydrogenase kinase-1, and pyruvate dehydrogenase, which are directly associated with aerobic glycolysis (283–285). The authors point out that the repeatedly observed impact of the CD44-ICD on HIF expression in SC suggests a more active role of CD44-ICD in SC maintenance and protection as previously anticipated (282).

In concern about the engagement of CD44 and its ligands in protection from oxidative stress, it also was described that neural SC reside undifferentiated in a HA rich matrix, but proliferate and differentiate upon hyaluronidase upregulation (286). Additional contributions of CD44 and HA on stress protection may be shared by HSC and LIC and are described below.

Taken together, HSC circumvent stress, which would drive them into proliferation and exhaustion, mostly by a minimal metabolic rate and the generation of energy via anaerobic metabolism. The main contribution of CD44 relies on the cotranscription factor activity of CD44-ICD.

### CD44, LIC, and apoptosis resistance

Besides contributing to circumvent stress, CD44 also actively promotes apoptosis resistance. From this activity of CD44, which does not appear to be of major importance for HSC stress resistance, LIC make profit. As to my knowledge, CD44-mediated apoptosis resistance proceeds in LIC and CIC via overlapping pathways, some examples of CIC will be included, where corresponding experiments have not yet been performed with LIC. There are two major mechanisms of CD44-mediated apoptosis protection: (i) initiation of signal transduction by CD44 crosslinking via HA, which frequently involves CD44v and associated RTK (287), and (ii) the crosstalk of CD44 with multidrug resistance genes that also is HA-dependent (288).

**Figure 4:**
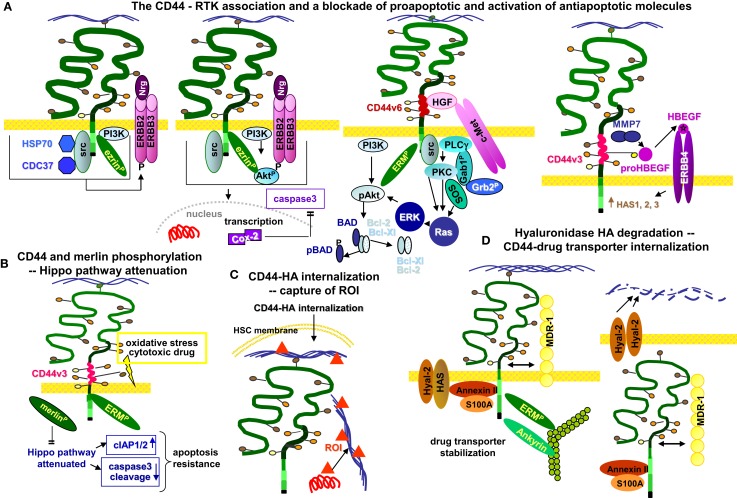
**CD44 interferes with apoptosis resistance in HSC/LIC**. **(A)** Four major pathways of RTK-promoted apoptosis protection are shown. (i) CD44–HA binding is accompanied by activation of CD44-associated src, ezrin phosphorylation, and PI3K activation, and assembly of a complex that includes, in addition, HSP70 and the co-chaperone CDC37, which promotes phosphorylation and activation of the ERBB2/ERBB3 RTK. (ii) The CD44–ERBB2/ERBB3 complex also provides a strong stimulus for Cox-2 transcription, which interferes with caspase3 activation. (iii) CD44v6 binds HGF and presents it to MET. Activation of the latter and the downstream signaling cascades require concomitant activation of CD44 associated pERM and src. Importantly, joining of signaling via the Ras/MAPK and the PI3K/Akt pathway that rely on the CD44 cytoplasmic tail can be initiated without any additional stimuli by a CD44v6-assembled matrix. (iv) CD44v3 binds proHBEGF, which becomes cleaved via CD44-associated MMP7. HBEGF binds and activates ERBB4. All of these CD44–RTK associations strongly promote activation of signaling cascades that block apoptosis and upregulate anti-apoptotic proteins. **(B)** Oxidative stress promotes merlin phosphorylation and dissociation from CD44. Merlin plays an important role in controlling the Hippo pathway. In CD44v expressing LIC, merlin is replaced by pERM, such that cIAP becomes increased and caspase3 activation does not take place, forcing LIC resistance toward cytotoxic drugs. **(C)** Particularly in HSC residing in the osteogenic niche, it has been observed that CD44-attached highMW HA can become internalized together with CD44, where HA acts as a catcher of ROI, which protects DNA from damage. **(D)** MDR proteins are associated with activated CD44 (highMW HA-ligated and associated with pERM). Instead, lowMW HA promotes CD44 and concomitantly MDR internalization, which is accompanied by a significant increase in cytotoxic drug sensitivity. Maintenance of the complex between CD44, pERM, actin, and MDR-1, as well as the recovery of the S100A/AnnexinII complex indicates internalization within caveolae and/or GEM. As far as LIC compete with HSC for the osteogenic niche, they will profit from the highMW HA matrix that via CD44 stabilizes MDR proteins within the cell membrane contributing to drug resistance.

Apoptosis resistance initiated by the cooperation of CD44- or CD44v with RTK mostly proceeds via activation of anti-apoptotic proteins. CD44 coimmunoprecipitates with all ERBB family members. The association of CD44 with ERBB2 and ERBB3 mediates heterodimerization and activation of the receptor in response to neuregulin, which strongly promotes CIC apoptosis resistance (289, 290). The impact of CD44 on ERBB2 activation is strikingly HA-dependent. CD44 crosslinking via HA initiates association of CD44 with ERBB2, which becomes phosphorylated. The complex, located in lipid rafts, includes ezrin, the chaperones HSP90 and CDC37, and PI3K, which accounts for drug resistance via activation of anti-apoptotic proteins. Apoptosis resistance is not seen when the HA–CD44 interaction is blocked, which causes complex disassembly and inactivation of ERBB2 (291). The authors also unraveled activation of an ERBB2-PI3K/Akt-β-catenin axis, which contributes to COX2 expression and COX2-promoted suppression of caspase3 activation. The data argue for a feedback loop, whereby COX2 strengthens HA production and promotes prostaglandin E2 expression (292). An additional feedback loop proceeds via formation of the ERBB2/ERBB4–CD44 complex, which via ERK activation promotes HA production by HAS1, -2, and -3 phosphorylation/activation (293) (Figure 4A).

Another, well-known pathway of apoptosis resistance involves the CD44v association with MET, which is initiated by HGF binding to CD44v3 or CD44v6 (138, 294), and promotes MET phosphorylation. MET phosphorylation requires the cytoplasmic tail of CD44 and the interaction with ERM proteins for activation of the Ras-MAPK pathway (294). CD44v6 binding to the ECM also activates the PI3K-Akt pathway and Wnt/β-catenin signaling (130, 295) and regulates *MET* transcription (86, 296). Similar observations account for insulin-like growth factor-1 receptor and PDGFR activation through HA-stimulated CD44 in transformed cells (133, 297, 298) (Figure 4A).

Importantly, CD44v6 promoted apoptosis resistance can also rely on the association of GEM-located CD44v6 with FAS. This association prevents trimerization of FAS upon ligand binding. Notably, apoptosis susceptibility strongly increases by a blockade of CD44v6 (299). Promoting FAS trimerization by a CD44v6 antibody blockade presents a new and interesting option in the therapy of CD44v6 expressing leukemia.

The interaction between CD44 and RTK can also proceed via proteases. CD44v3 binds the proform of the heparin binding epidermal growth factor (HB-EGF), which is cleaved by CD44-recruited MMP7. Cleaved HB-EGF binds and activates ERBB4, which signals for cell survival (300, 301). The interaction of CD44 with MMP9 leads to apoptosis protection independent of RTK. CD44 and MMP9 expression are interdependent (149), and in CLL patients with poor prognosis CD44, CD49, and MMP9 are physically associated (302, 303). CD44-associated MMP14 accounts for proMMP9 cleavage (191). Activated MMP9 can interfere with TGFβ activation, whereby several mechanisms of TGFβ-promoted apoptosis become silenced (304, 305).

An additional pathway of CD44-promoted resistance to reactive oxygen- and cytotoxic drug-induced stress under physiological and pathological conditions proceeds via the mammalian Hippo signaling pathway. In the resting state, CD44-associated merlin accounts for JNK, p53, and p21 upregulation, and YAP as well as ciAP1/2 downregulation, which jointly promote caspase3 activation and apoptosis. When CD44 becomes activated by HA binding, merlin is phosphorylated and dissociates from CD44. In the absence of merlin, CD44 directly regulates YAP expression via active RhoA. Thereby, the HIPPO pathway becomes blocked, which results in increased apoptosis resistance (306, 307)^.^ In a feedback loop, activated YAP binds to the promoter of RHAMM, thereby inducing RHAMM transcription (308, 309) (Figure 4B).

Besides via CD44-associating molecules, the direct interaction between CD44 and HA strongly affects apoptosis resistance. Exploring the effect of HA on the extent of DNA damage induced by exogenous and endogenous oxidants revealed that CD44 in SC internalizes HA by endocytosis. One of the functions of the internalized HA is the protection of DNA from oxidants. The authors propose entrapment of iron ions. Thereby the Fenton’s reaction, which produces secondary oxidative species becomes inhibited. Alternatively, though not mutually exclusive, HA directly scavenges primary and secondary ROI, which results in intracellular HA degradation (310). Palmitoylation and GEM recruitment of CD44 is a precondition for HA internalization (311). Thus, attenuating oxidant-induced damage can proceed through direct scavenging of oxidant molecules by HA (312) (Figure 4C).

Drug resistance can also be promoted through CD44v3–HA binding, which via the Oct4-Sox2-Nanog complex induces miR-302 transcription (74). The CD44–HA induced nuclear translocation of Nanog also leads to miR-21 production and upregulation of apoptosis inhibitors and MDR1 (77, 313).

Last, the interplay between CD44 and HA accounts for rapid drug elimination via drug transporters (288), which creates a major obstacle in leukemia and cancer therapy (314). Both CD44 and HA contribute to drug resistance. MDR genes are associated with CD44 and CD44 regulates expression of drug transporters. This likely is due to HA-activated CD44 binding to Gab1, which promotes PI3K activation. Activated PI3K stimulates HA production as well as MDR transporter expression (315, 316). Alternatively, though not mutually exclusive, HA binding to CD44 up-regulates p300 expression and its acetyltransferase activity. This, in turn, promotes acetylating β-catenin and NFκB-p65. Activated β-catenin and NFAT act as cotranscription factors with NFκB in MDR1 transcription (317). The direct involvement of HA was demonstrated by replacing high MW by low MW HA. In the presence of high MW, HA activated CD44 is predominantly recovered in GEM and is associated with ERM and actin. MDR1 is associated with CD44 and the association stabilizes MDR1 expression. Instead, low MW HA does not stabilize the complex, but rather supports internalization such that all components of the complex including S100A and Annexin II are recovered in the cytoplasm (318). Whether the internalized complex becomes degraded or released as exosomes has not been explored. Independent of the answer to this question, the reduction of MDR1 in the cell membrane is accompanied by increased drug susceptibility (319). Notably, the three hyaluronan synthases are supposed to produce HA of different size. However, this as well as the major transcription factors engaged in HAS1, -2, and -3 transcription remain to be defined. Transcriptional regulation of hyaluronidases also awaits unraveling (320, 321). Taking into account that hyaluronidase as well as small HA oligosaccharides can improve drug efficacy (318), and that HA-CD44 cross-linking regulates expression of drug transporters (315, 316), filling this gap becomes demanding to improve therapeutic targeting of HA [review in Ref. (322, 323)] (Figure 4D).

The major importance of CD44 in apoptosis resistance relies (i) on the association of activated (HA-crosslinked) CD44/CD44v with RTK, which promote activation of anti-apoptotic signaling cascades, (ii) on interferences of CD44v6 with FAS trimerization, and (iii) the engagement of CD44 in regulating drug-resistance gene expression. The impact of CD44 is efficiently reinforced by high MW, but not low MW HA, where the latter may open a therapeutic window.

## Open Questions

This review highlights the importance of CD44 in the crosstalk between HSC/LIC and the surrounding matrix. However, due to space constraints, this review does not cover the role of CD44 in all aspects of hematopoiesis and leukemia induction; this can be found within an excellent review which also focuses on signal transduction and transcription (324). Furthermore, while CD44-based therapeutic concepts have been highlighted in individual sections within this review, there is some excellent literature which describes CD44 antibody and vaccination-based therapeutic concepts that may be of further interest to the reader (325–332). However, there are a number of key issues/questions that need to be addressed before a major therapeutic breakthrough can be achieved:

1. More information on the active contribution of the niche is required as well as the contribution of the individual components. This includes the possible transfer of information, comprising miRNA, via exosomes and accounts for HSC and LIC.
2. There is insufficient information on the modulation of the osteogenic niche by LIC, to safely protect HSC, if LIC was to be targeted, and to reconstruct a destroyed niche for unimpaired hematopoiesis.
3. The generation of LIC is not well understood. Again, the possibility has to be taken into account that LIC are instructed by surrounding cells, preferably HSC or MSC, via exosomes. Furthermore, there is a paucity of knowledge on the decision for self-renewal versus differentiation, which to some degree also accounts for HSC.
4. Much progress has been made in homing, migration, and signal transduction, which could well become the first question to be comprehensively answered.

In summary, the specific therapeutic targeting of LSC is still very much a field in its infancy (323). However, there is justified hope that this may change in the near future.

## Conclusion

Stem cells require a niche, which has been particularly well explored for HSC, where the central importance of the CD44–HA interaction, including more recently the cellular stroma elements, is amply demonstrated. The CD44–HA crosstalk promotes adhesion and via cytokines/chemokines harbored in the BM-Str, homing, and migration of HSC as well as HSC quiescence and resistance to low oxygen pressure. LIC share with HSC the requirement for the crosstalk with the stroma to promote adhesion, homing, and migration. Apoptosis resistance of LIC, though strikingly dependent on the CD44-HA crosstalk, proceeds differently to that of HSC predominantly via CD44v and the cooperation with RTK, proteases, and drug transporters.

The abundant array of HA-bound CD44-initiated activities relies on the cooperation of CD44 with multiple membrane molecules including integrins, chemokine receptors, RTK, and proteases as well as its transient association with cytoskeletal linker molecules and cytoplasmic signal transducers, which includes central stem cell fate regulators. The multitude of interactions is fostered by the GEM location of CD44, which also promotes the proximity to proteases. Proteases facilitate CD44 cleavage, where the CD44-ICD acts as a cotranscription factor. It has been suggested, but needs further approval, that CD44-ICD also regulates miR transcription/repression of CD44 cooperation partners, thereby creating an additional feedback loop.

In view of the most promising results in leukemia therapy by blocking CD44, awareness increased on possible selective differences between HSC and LIC in the crosstalk with the osteogenic niche. Several elegant and sophisticated studies clearly demonstrated the existence of differences not only between HSC and LIC but also between distinct leukemia. Progress in this field will greatly facilitate selective therapeutic interference with LIC homing and may allow for corrections of the LIC-distorted osteogenic niche. The latter as well as the interaction with the vascular niche and homing into the osteogenic niche is of particular interest in view of the HSC transfer being frequently a last chance for curative therapy. Further clarifying HA production and degradation may also open new avenues for a therapeutic dissection between the HSC- and the LIC–HA crosstalk. Last, not least, uncovering the importance of miRNA and the role of exosomes in miRNA transfer will add optimizing the HSC–HA crosstalk in the osteogenic niche and will allow interfering with niche destructing activities of LIC.

## Conflict of Interest Statement

The author declares that the research was conducted in the absence of any commercial or financial relationships that could be construed as a potential conflict of interest.

## Abbreviations

ASC, adult stem cells; bFGF, basic fibroblast growth factor; BM, bone marrow; BMP, bone morphogenetic protein; BM-Str, BM stroma cells; C, complement; CD44s, CD44 standard isoform; CD44v, CD44 variant isoforms; CIC, cancer initiating cells; ECM, extracellular matrix; ERM, ezrin, radixin, moesin; ESC, embryonic SC; FN, fibronectin; GAG, glucosaminoglycan; GEM, glycolipid enriched membrane microdomains; HA, hyaluronic acid; HAS, hyaluronan synthase; HGF, hepatocyte growth factor; HSC, hematopoietic SC; ICD, intracellular domain; kd, knockdown; ko, knockout; LIC, leukemia initiating cells; MSC, mesenchymal SC; Mϕ, macrophages; OPN, osteopontin; PCCD4, programmed cell death 4; PDGFR, platelet-derived growth factor receptor; RTK, receptor tyrosine kinase; TGF, transforming growth factor; TPO, thrombopoietin; VEGF, vascular endothelial growth factor; wt, wild type.
